# Suitability of Mycelium-Reinforced Nanofiber Mats for Filtration of Different Dyes

**DOI:** 10.3390/polym15193951

**Published:** 2023-09-29

**Authors:** Angela Heide, Philip Wiebe, Lilia Sabantina, Andrea Ehrmann

**Affiliations:** 1 Faculty of Engineering and Mathematics, Bielefeld University of Applied Sciences and Arts, 33619 Bielefeld, Germany; 2 Faculty of Clothing Technology and Garment Engineering, School of Culture + Design, HTW Berlin—University of Applied Sciences, 12459 Berlin, Germany; lilia.sabantina@htw-berlin.de

**Keywords:** needleless electrospinning, dye filtration, molecular weight, mushroom mycelium

## Abstract

Electrospun nanofiber mats have a high specific surface area and very small pores which can be tailored by the spinning process. They are thus highly suitable as filters for small particles and molecules, such as organic dyes. On the other hand, they are usually very thin and thus have low mechanical properties. As a potential reinforcement, mycelium of *Pleurotus ostreatus* was grown on poly(acrylonitrile) nanofiber mats and thermally solidified after fully covering the nanofiber mats. This study investigates whether the filtration efficiency of the nanofiber mats is altered by the mycelium growing through it and whether the mechanical properties of the nanofibrous filters can be improved in this way. The study shows fast and reliable growth of the mycelium on the nanofiber mats and high filtration efficiency for astra blue and chlorophyll, while indigo carmine showed only very low filtration efficiency of up to 20%. For chlorophyll and safranin, membranes with mycelium showed higher filtration than pure nanofiber mats. In diffusion cell tests, especially astra blue was strongly adsorbed on the membranes with mycelium.

## 1. Introduction

The electrospinning technique allows the production of nanofibers with diameters in a typical range of tens to hundreds of nanometers from diverse polymers [1,2,3]. Besides needle-based setups in which a syringe with a fine needle introduces a polymer solution or a polymer melt into a strong electric field, several needleless setups have been reported in the last years, such as wires, rotating drums, and multiple nozzles, etc. [4,5,6]. In all geometries, the electric field forces the polymer solution to form a Taylor cone before part of the droplet is released towards the counter electrode, during this process being stretched and dried [7,8,9].

Generally, a broad range of materials can be electrospun, from single polymers to polymer blends to polymers with embedded nanofibers, to produce nanofibrous membranes with the required physical and chemical properties [10,11,12]. On the other hand, some polymers have special properties making them advantageous for electrospinning. Poly(acrylonitrile) (PAN) belongs to these often used materials since it is highly suitable for subsequent carbonization and, often more important, spinnable from the low-toxic solvent dimethyl sulfoxide (DMSO) [13,14].

Electrospun nanofiber mats from different polymers and additions can be used for a broad variety of applications, such as biomedical applications [15,16], batteries [17], air filtration [18,19] or water filtration [20,21,22]. For most of these applications, the mechanical properties of the nanofibrous membranes play an important role. Especially for water filtration, it is necessary to combine sufficient mechanical properties with the required water permeability, if filtration through the membrane is planned, while nanofibrous membranes for adsorption can also be mounted on a rigid substrate [23]. Typically, a porous supporting layer is applied to stabilize the nanofiber mat mounted on it, which does not impede the nanofiber mat’s transport properties [24,25]. Other suggestions found in the literature are related to additives, cross-linking, fiber welding [26], or even 3D printing of a support layer [27]. In all these cases, the nanofiber mat can directly be electrospun on the support layer, which usually causes the problem that the membrane shrinks during drying and thus may break or delaminate; it can be glued on a substrate, necessitating additional glue which will influence filtration; or it can be sandwiched without full-surface area adhesion, which does not impede breaks in the nanofiber mat due to shear forces. Thus, a substrate which adheres to the nanofiber mat automatically would be an interesting alternative.

Here we investigate the suitability of a biological support in the form of mushroom mycelium. Previous studies have shown that the mycelium of different fungi can grow on textile substrates [28] and even on electrospun nanofiber mats [29,30,31]. A well-suited mushroom for such experiments is *Pleurotus ostreatus* which is edible and contains a large amount of chitin in the mycelium cell walls, making it mechanically stable [32]. On the other hand, it is not yet known whether the mycelium growing on the nanofiber mat will produce holes in the membrane which impede the required filtration properties. This study thus compares PAN nanofiber mats with and without thermally treated *P. ostreatus* mycelium grown on it as filters, using dyes of different molecular weights as model substances and testing filtration through the nanofibrous membranes as well as adsorption on it.

## 2. Materials and Methods

### 2.1. Electrospinning

Spinning solutions were prepared by dissolving 16% PAN (X-Pan, copolymer with 6% methyl methacrylate, from Dralon, Dormagen, Germany) in DMSO (min 99.9%, S3 Chemicals, Bad Oeynhausen, Germany) by stirring with 200/min at room temperature for 48 h. The needleless electrospinning machine “Nanospider Lab” (Elmarco Ltd., Liberec, Czech Republic) was applied to prepare nanofibrous membranes on a polypropylene (PP) nonwoven substrate. The following spinning parameters were used: nozzle diameter of 0.8 mm, voltage of 80 kV, resulting in a current of 0.05–0.07 mA, static substrate, distance between bottom electrode and substrate of 240 mm, distance between ground electrode and substrate of 50 mm, temperature in the spinning chamber of 20 °C, and relative humidity of 33%. Spinning was performed for 40–50 min.

The middle parts of the nanofiber mats were cut into round segments with a diameter of 6 cm. The mass of each specimen was measured by an analytical balance VWR LA Classic (VWR International GmbH, Darmstadt, Germany). Even in the case of optically homogeneous nanofiber mats, a study has shown strong deviations in areal weight and thickness between neighboring segments [33], making it necessary to measure each single sample. To investigate the influence of the areal weight, thicker and thinner areas of the produced nanofiber mats were chosen, resulting in a broad range of areal weights of about 0.9 g/m²–13.9 g/m². All in all, 178 nanofiber mat samples were cut and measured, half of which were inoculated with mushroom mycelium, while the other half was tested as pure nanofibrous membranes.

### 2.2. Sterilization of the Nanofiber Mats

In order to sterilize the nanofiber mats before inoculation with the mushroom, they were ozonized for 80 min and afterwards put into zip-locked bags inside the ozone box to avoid re-contamination.

### 2.3. Preparation of Agar Plates

As the nutrient medium for mycelium growth, malt extract agar was chosen. It was produced by 0.75 L of deionized water, 18 g of agar (agar–agar Kobe I, Roth, Karlsruhe, Germany), 15 g of barley malt extract (Lindenmeyer GmbH & Co. KG, Weinsberg, Germany) and 0.75 g of peptone water Fluka Analytical, Charlotte, NC, USA). Afterwards, the fluid solution was autoclaved and poured into autoclaved 8 cm diameter petri dishes (VWR, Darmstadt, Germany).

### 2.4. Inoculation with Liquid Mycelium and Mycelium Cultivation

After the cut nanofiber mats were placed on the agar plates, half of them were inoculated with sterile liquid mycelium “Liquid Pure Culture Pleurotus ostreatus MG1005” and “Liquid Pure Culture Pleurotus ostreatus var. columbinus MG1010” (abbreviated as *P. ostreatus blue*), respectively (MycoGenetics Pilz-Shop, Everswinkel, Germany), using 3 drops (partly 5 drops, as discussed below) of liquid mycelium culture per petri dish, and afterwards sealed with Parafilm (Pechiney Plastic Packaging, Chicago, IL, USA).

**Figure 1:**
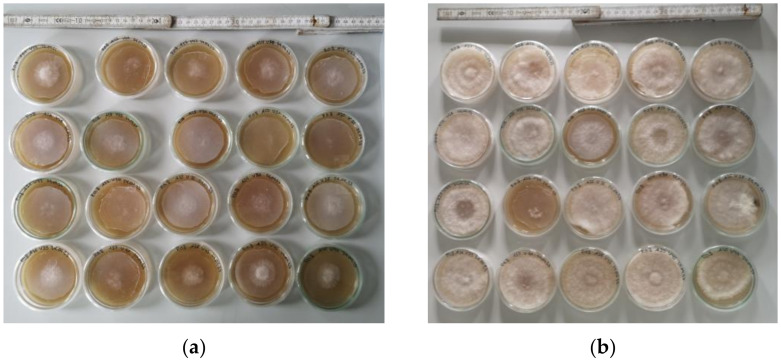
Growth of the mycelium of *P. ostreatus blue*: (**a**) day 3 after inoculation; (**b**) day 8 after inoculation.

Mycelium cultivation occurred for 21 days in the dark at temperatures of 21–22 °C and a relative humidity of 42–60%. Mycelium growth was observed by photographic images from a constant distance which were analyzed software ImageJ 1.51j8 (from National Institutes of Health, Bethesda, MD, USA) by counting the pixels in the mycelium area and calculating an area and a corresponding diameter from this value. Figure 1 exemplarily depicts the growth of *P. ostreatus blue* on day 3 and day 8 after inoculation, respectively.

Half of the samples with *P. ostreatus* and *P. ostreatus blue* mycelium, respectively, were thermally treated at 60 °C for 6 h to inactivate the mushroom.

### 2.5. Filtration

The dye solutions used for filtration tests were astra blue 0.025%, safranin 0.0125%, indigo carmine 0.0025%, and chlorophyll 0.025%.

Parts of the experiments were performed using a side-bi-side diffusion cell (PermeGear, Hellertown, PA, USA). In these cells, a membrane is placed between both cell halves, and both cell halves are continuously stirred. In the recent study, parts of the samples were cut to be used as membrane separating both cell halves, where the mycelium side was oriented to the left side. The left cell half was filled with tap water, the right one with dye solution, both cell halves were closed, and the cell was activated. After 24 h, 2 mL were pipetted out of each cell half for further investigation.

The other experiments were carried out by using a vacuum pump (CVC 3000, vacuubrand, Wertheim, Germany) to pump dye solution through the sample (with the mycelium at the bottom) placed on a Büchner funnel into an Erlenmeyer flask, applying an underpressure of 2 mbar. After filtration, the samples were stored in the dark to avoid bleaching.

A UV/Vis spectrometer Genesys 10S (Thermo Scientific, Dreieich, Germany) was used to measure the absorbance of the samples before and after filtration in the wavelength range from 300 to 800 nm.

**Figure 2:**
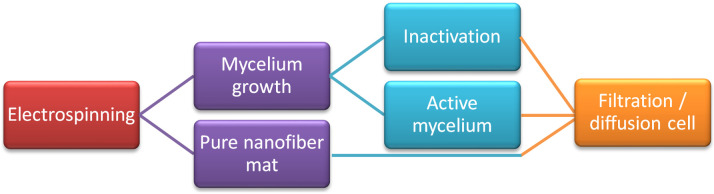
Schematic of the research methodology.

The process is schematically depicted in Figure 2.

## 3. Results and Discussion

### 3.1. Mycelium Cultivation

**Figure 3:**
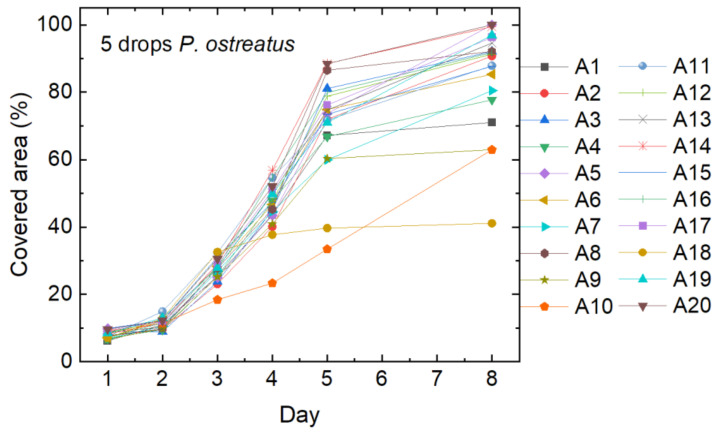
Cover factor of the *P. ostreatus* mycelium grown on agar plates with nanofiber mats on top after inoculation with 5 drops of liquid mycelium.

The results of the mushroom mycelium growth, started with five drops of liquid mycelium of *P. ostreatus*, is shown in Figure 3. Sample names correspond to an internal nomenclature and are only given for further discussion. Most of the 20 samples showed suitable growth and covered the whole agar plate with nanofiber mat on top after 8 days. On some plates, however, not the whole plate was covered (e.g., A1, A10), and in some cases, growth has apparently stopped after some days (e.g., A18). The samples which were not fully covered after the full growth period of 21 days were not used in subsequent filtration tests.

**Figure 4:**
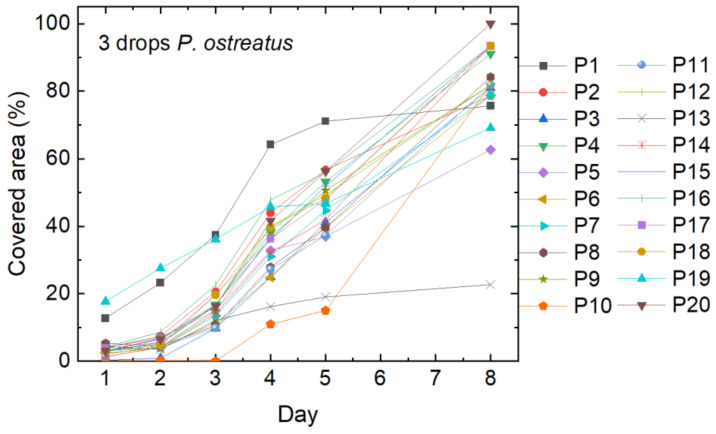
Cover factor of the *P. ostreatus* mycelium grown on agar plates with nanofiber mats on top after inoculation with 3 drops of liquid mycelium.

On the agar plates with nanofiber mats on which only three drops of liquid mycelium were used for inoculation, the coverage of the nanofibrous membrane was much slower, as Figure 4 shows. On one of the samples (P13), mycelium growth stopped after around five days, while it was accelerated in sample P10 at this time. Most of the samples, however, were fully covered with mycelium after the full growth period of 21 days.

**Figure 5:**
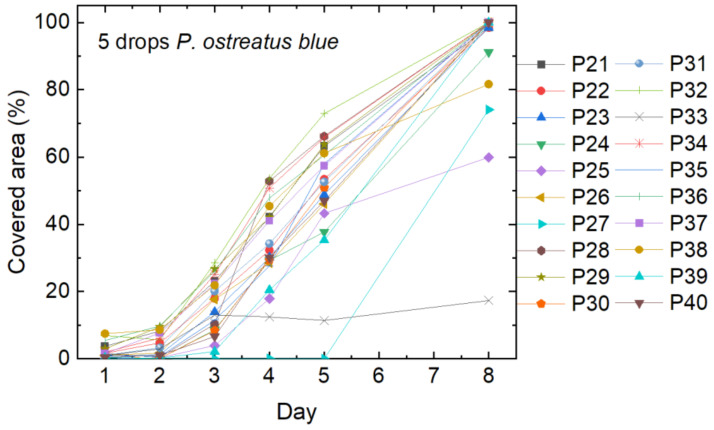
Cover factor of the *P. ostreatus blue* mycelium grown on agar plates with nanofiber mats on top after inoculation with 5 drops of liquid mycelium.

Next, *P. ostreatus blue* was inoculated with five drops per petri dish. The results are depicted in Figure 5. The curves look similar to the growth curves of *P. ostreatus*, as depicted in Figure 3, with a generally lower cover factor on day 5, but more samples reaching 100% on day 8. On one of the samples (P33), the mycelium stopped growing after 3 days, while another sample (P27) only starting growing after 5 days.

**Figure 6:**
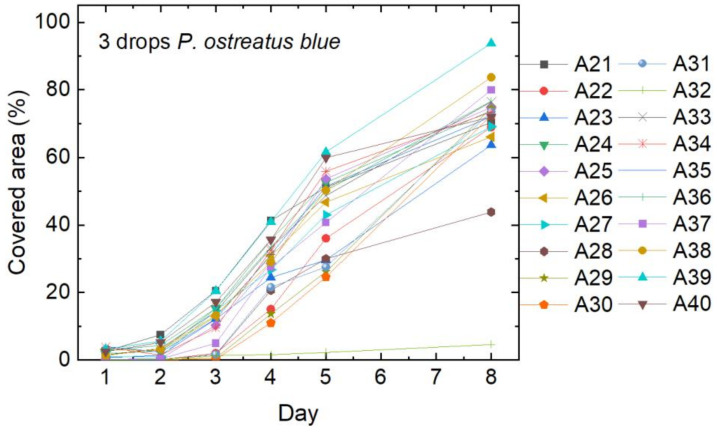
Cover factor of the *P. ostreatus blue* mycelium grown on agar plates with nanofiber mats on top after inoculation with 3 drops of liquid mycelium.

Finally, Figure 6 shows the growth of *P. ostreatus blue* after inoculation with three drops of the respective liquid mycelium. Only one sample (A32) does not show any mycelium growth. On average, the growth curves are very similar to Figure 4, showing that both strains of *P. ostreatus* grow well.

### 3.2. Filtration through the Nanofiber Mats

**Figure 7:**
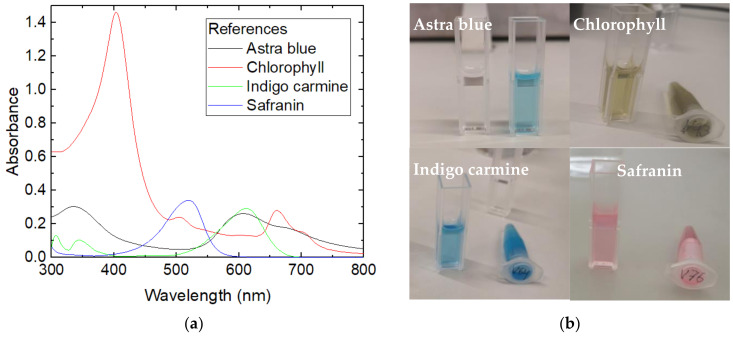
(**a**) Reference spectra of the four dyes under investigation; (**b**) optical appearance of the dye solutions.

The spectra of the dye solutions used in all filtration tests are depicted in Figure 7. For chlorophyll, the spectrum shows the usual two peaks around 400 nm and 660 nm, resulting in a green appearance [34]. Astra blue appears blue, as its name indicates, while safranin has a reddish look, and indigo carmine is also blue. For the evaluation of the filtration efficiency, the following wavelengths were investigated: 660 nm for chlorophyll, 600 nm for astra blue, 520 nm for safranin, and 612 nm for indigo carmine.

The molecular weights of the dyes are typically in the range of 0.9 kDa for chlorophyll [35,36], 1.0 kDa for astra blue [37], 0.35 kDa for safranin [38], and 0.47 kDa for indigo carmine [39], suggesting that chlorophyll and astra blue should be filtered better than safranin and indigo carmine by identical nanofibrous filters.

**Figure 8:**
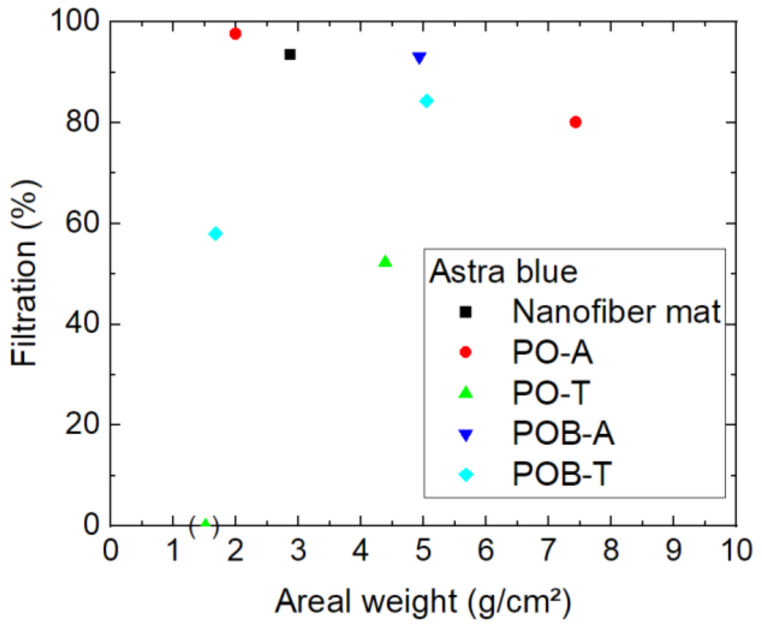
Filtration efficiency of astra blue dye through different nanofiber mats with and without mycelium. The codes denote PO—*P. ostreatus*, POB—*P. ostreatus blue*, A—alive, T—thermally treated, i.e., inactivated. Brackets mark samples with problems observed, e.g., defects occurring during filtration.

Next, Figure 8 depicts the results of filtration through different nanofiber mats, with and without mushroom mycelium, tested by astra blue, the dye with the highest molecular weight. The codes marking the different samples are given in the caption of Figure 8.

The sample (PO-T) with a filtration efficiency of 0% must be excluded from the evaluation since this very thin sample, recognizable by its low areal weight, could not be fully attached along the whole surface area of the Büchner funnel so that the dye solution had the possibility to reach the Erlenmeyer flask by surrounding the filter. The measurement point is nevertheless shown in Figure 8 to mention this problem.

The main question of whether the mycelium punctuates the nanofibrous membranes so that their filtration efficiency is reduced can be neglected for astra blue, as there are two samples with mycelium (PO-A and POB-A) with similar filtration efficiency above 90%. Comparing all results from this figure, however, raises a new question, i.e., whether thermal treatment of the samples to inactivate the mycelium may damage the samples, since both relatively low filtration efficiencies with values around 50–60% are related to thermally treated samples. On the other hand, it must be mentioned that there is no positive correlation between filtration efficiency and areal weight visible, as it could be expected. This finding shows, at least for the largest of the dye molecules under examination, that the filtration efficiency depends in a more complex way on the nanofibrous filters.

**Figure 9:**
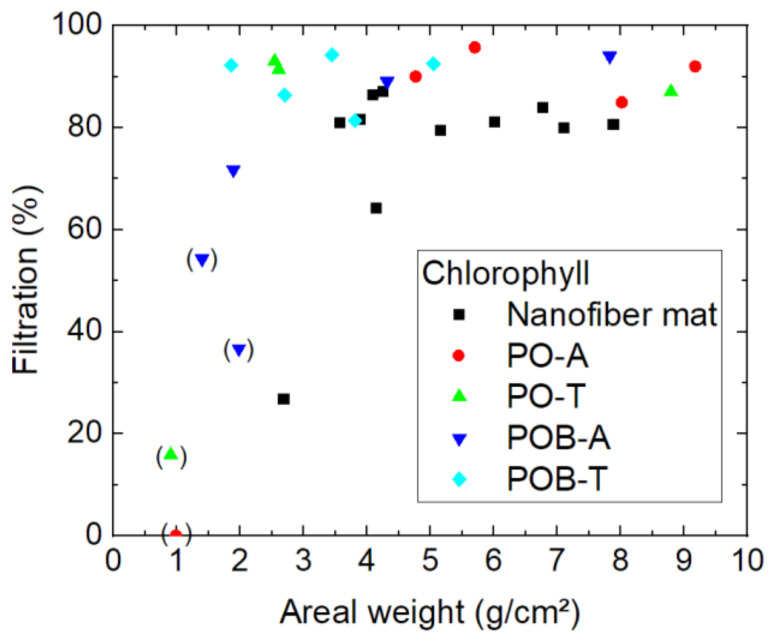
Filtration efficiency of chlorophyll dye through different nanofiber mats with and without mycelium. The codes denote PO—*P. ostreatus*, POB—*P. ostreatus blue*, A—alive, T—thermally treated, i.e., inactivated. Brackets mark samples with problems observed, e.g., defects occurring during filtration.

Filtration of the next-smallest molecule, chlorophyll, is depicted in Figure 9. To enable distinguishing between different kinds of sample better, more specimens were tested according to chlorophyll filtration than in the first test series with astra blue.

Here again, some samples had to be removed from the evaluation, this time due to visible breaks inside the nanofiber mat which occurred during filtration (marked by brackets). It can be speculated that the pure nanofiber mat with an areal weight of 2.7 g/m² and a filtration efficiency of 27% was either also broken or not perfectly mounted at the boarders, since none of the other samples shows similarly low filtration values. Most of the other samples reach values around 80–100%. Interestingly, there is again no correlation between the mass per unit area and the filtration efficiency visible. Instead, the lower filtration values for some of the very thin nanofiber mats can in most, if not all cases, be attributed to gaps in the nanofiber mats or along their borders, since these fine membranes are hard to handle.

On the other hand, it should be mentioned that filtration through the pure nanofiber mats shows a slightly lower filtration efficiency than most membranes with mycelium grown on them. However, this effect was not observed for astra blue which has a similar molecular weight, and the sample size is still not very large, so that this potential effect has to be investigated in more detail in the future.

**Figure 10:**
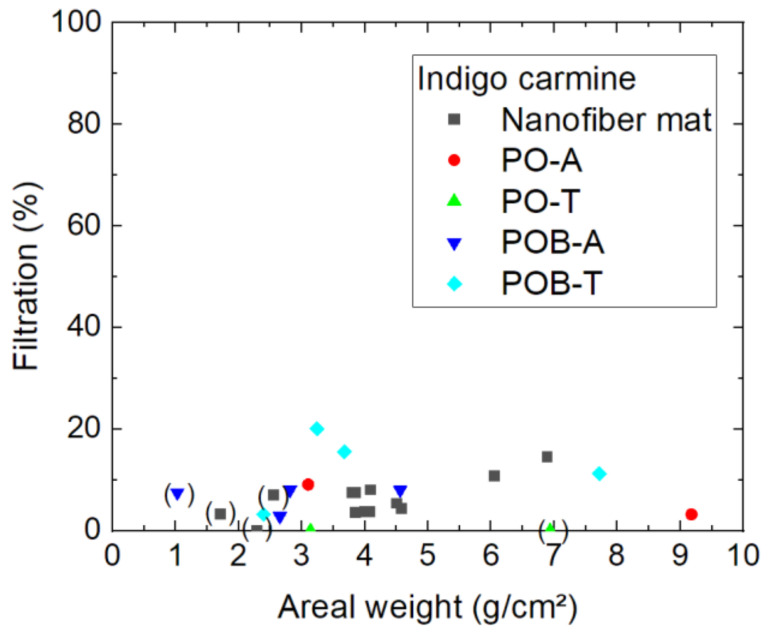
Filtration efficiency of indigo carmine dye through different nanofiber mats with and without mycelium. The codes denote PO—*P. ostreatus*, POB—*P. ostreatus blue*, A—alive, T—thermally treated, i.e., inactivated. Brackets mark samples with problems observed, e.g., defects occurring during filtration.

Going further with the smaller molecules, Figure 10 shows the efficiency of indigo carmine filtration. As directly visible, the general filtration efficiency is significantly lower than for both larger molecules. Not only for the samples with visible damages, as marked by brackets, the efficiency is generally lower than approx. 20%. This shows clearly that the nanofiber mats produced with the aforementioned spinning parameters are not able to filter such small molecules, independent of the areal weight. For such purposes, the spinning parameters must be modified accordingly.

**Figure 11:**
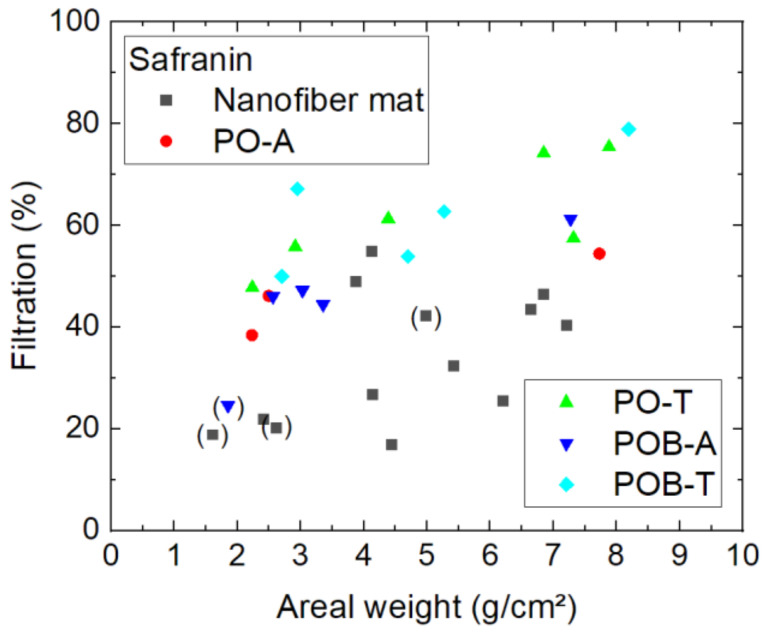
Filtration efficiency of safranin dye through different nanofiber mats with and without mycelium. The codes denote PO—*P. ostreatus*, POB—*P. ostreatus blue*, A—alive, T—thermally treated, i.e., inactivated. Brackets mark samples with problems observed, e.g., defects occurring during filtration.

The smallest molecule, safranin, shows an unexpected behavior in the filtration tests, as visible in Figure 11, by having significantly higher filtration efficiency than indigo carmine. A possible explanation for this finding is the cationic nature of safranin, as opposed to the anionic indigo carmine, which increases its electrostatic interaction asan adsorbent and thus the adsorption capacity for a neutral pH value [40]. Whether this effect is responsible for the unexpectedly high filtration efficiency of safranin has to be tested in a future experiment.

On the other hand, here again it is visible that the nanofiber mats with additional mushroom mycelium grown on them, especially in the inactivated (thermally treated) form, show on average a higher filtration efficiency than the pure nanofiber mats. The advantageous behavior of the inactivated mycelium, as compared to the activated one, has not been observed for chlorophyll filtration (Figure 9); however, there the efficiency values are generally larger, so that such an effect may stay unobserved. Since the apparently broken samples are mostly pure membranes without mycelium, a potential assumption is that the pure nanofiber mats often have fine damages and that the support of the mycelium avoids such unobserved small fractures. On the other hand, it is also possible that adsorption of safranin on mycelium supports the filtration effect.

### 3.3. Filtration in a Diffusion Cell

**Figure 12:**
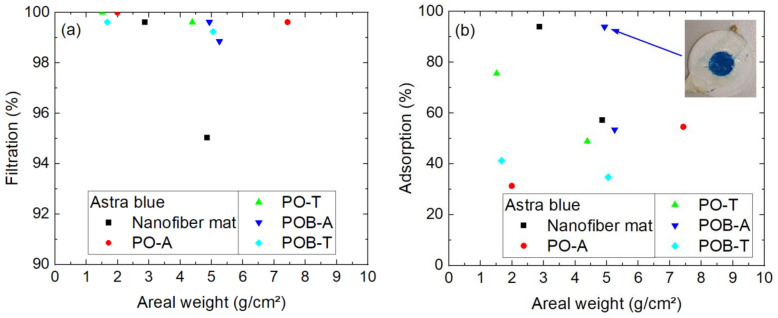
Results of 24 h diffusion cell measurements with astra blue: (**a**) filtration efficiency; (**b**) adsorption of dye on the membrane. Inset: POB-A sample with 4.9 g/m² after 24 h in the diffusion cell. The y-scales differ.

While the filtration tests by pumping a dyed solution through a nanofiber mat are mostly influenced by the pore sizes, but potentially also by adsorption of the dye molecules on the membranes, as discusses before, the diffusion cell enables diffusion testing without an applied pressure and the adsorption on the membrane. The results for diffusion tests with astra blue are presented in Figure 12. As Figure 12a shows, the filtration efficiency regarding diffusion from one cell half into the other is for most samples near to 100%, i.e., nearly no dye is able to diffuse through the membrane. The adsorption on the nanofiber mat including the mycelium, as calculated from the “missing” dye measured in both cell halves after the test duration of 24 h, is depicted in Figure 12b. Here, no clear differences are visible between the different samples with and without mycelium nor regarding the areal weight of the samples. Some of the samples, like the one depicted in the inset, show a very high adsorption which is also optically well visible.

**Figure 13:**
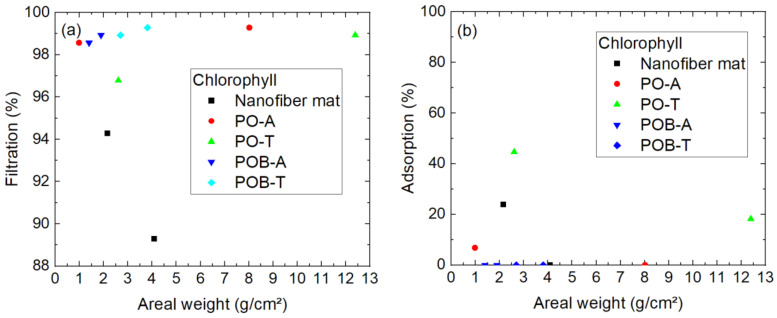
Results of 24 h diffusion cell measurements with chlorophyll: (**a**) filtration efficiency; (**b**) adsorption of dye on the membrane. The y-scales differ.

Chlorophyll as the next-smaller molecule showed slightly higher filtration efficiency for membranes with mushroom mycelium, as compared to pure nanofiber mats (Figure 9). A similar effect is visible in Figure 13a, showing the filtration efficiency in the diffusion cell, where most membranes including mycelium show an efficiency around 98–99%, while the values of the pure nanofiber mats are lower. Apparently, this cannot be attributed to different adsorption on the membranes with and without mycelium, as Figure 13b shows. Most samples showed no adsorption, i.e., no loss of dye during the experiment. In cases where the dye concentration seems to have even slightly increased during the time of the experiment, most probably due to small measurement deviations, the adsorption values are set to 0% in Figure 13.

**Figure 14:**
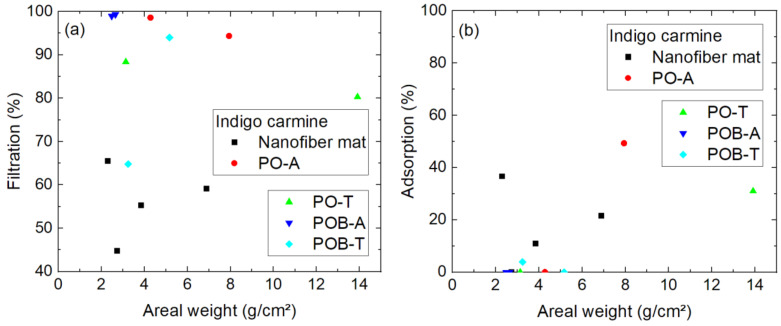
Results of 24 h diffusion cell measurements with indigo carmine: (**a**) filtration efficiency; (**b**) adsorption of dye on the membrane. The y-scales differ.

Indigo carmine was the dye with by far lowest filtration efficiency in the tests using a pump. In the diffusion tests, the filtration efficiency is also significantly lower than for both larger dye molecules (Figure 14a). Especially, the pure nanofiber mats show only filtration efficiencies around 44–65%, while most of the membranes with mycelium result in much higher efficiencies. Outliers like the POB-T sample with an areal weight of 3.2 g/m² can most likely be attributed to unrecognized damages of the samples, as discussed in the previous section.

The adsorption is for most samples near to 0%, while three of the four pure nanofiber mats as well as two of the specimens with *P. ostreatus* mycelium have measurably adsorbed the dye (Figure 14b).

**Figure 15:**
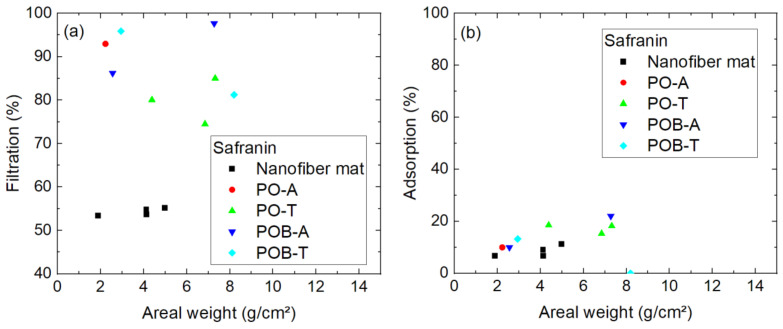
Results of 24 h diffusion cell measurements with safranin: (**a**) filtration efficiency; (**b**) adsorption of dye on the membrane. The y-scales differ.

Finally, Figure 15 shows the diffusion test results of safranin, which showed better filtration in the pumped experiment than indigo carmine. Here, the filtration efficiency is in the range of 50–55% for all pure nanofiber mats (Figure 15a) and between 74% and 98% for the nanofiber mats including mycelium, i.e., not clearly enhanced as compared to filtration of indigo carmine. This advantage of the samples with mycelium was also recognized in the aforedescribed filtration tests.

The previous speculation that safranin might show high adsorption on the mycelium, however, is not fully supported by the adsorption measurements in the diffusion cell (Figure 15b), where only slightly increased adsorption was measured for the average membrane with mycelium, as compared to the pure nanofiber mats.

**Figure 16:**
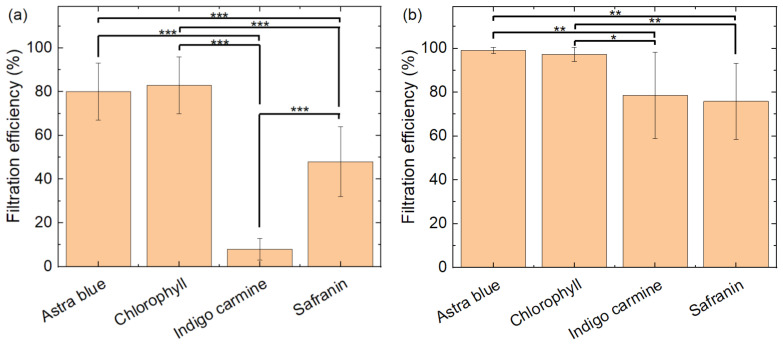
Comparison of filtration efficiency for different dyes (**a**) through the membrane; (**b**) in the diffusion cell. Error bars indicate standard deviations. Statistical differences are denoted as * (*p* < 0.05), ** (*p* < 0.01), or *** (*p* < 0.001), respectively.

Figure 16 shows the comparison of the filtration efficiency through a nanofiber mat (Figure 16a) and in a diffusion cell (Figure 16b) for the four dyes under examination in this study. Statistical differences are calculated by the Welsh test. As described before, filtration of astra blue and chlorophyll is very similar in both test procedures, while indigo carmine and safranin show significantly lower filtration. A significant difference between indigo carmine and safranin is only visible for the filtration through the nanofiber mats (Figure 16a).

**Figure 17:**
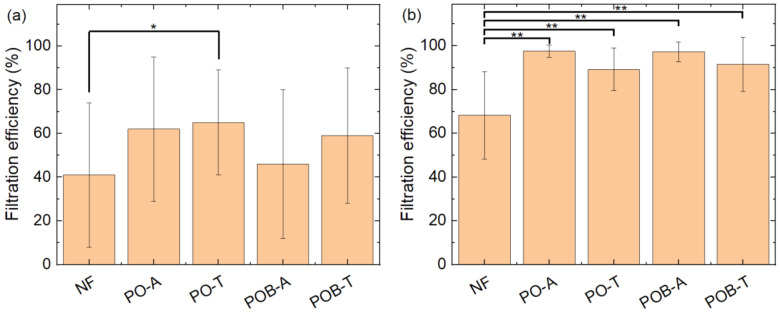
Comparison of filtration efficiency for different nanofiber mats/composites (**a**) through the membrane; (**b**) in the diffusion cell. Error bars indicate standard deviations. Statistical differences are denoted as * (*p* < 0.05), ** (*p* < 0.01), or *** (*p* < 0.001), respectively.

A comparison of the pure nanofiber mat with the membranes with additional mycelium only shows a slightly significant difference in the pumped experiment between the pure membrane (NF) and the nanofiber mat with thermally treated *P. ostreatus* (PO-T) (Figure 17a). In the diffusion cell test, all membranes with mycelium show a significantly higher filtration efficiency than the pure nanofiber mat (Figure 17b).

Comparing the results of both experiments, it can be stated that both larger dye molecules are generally filtered with a high efficiency, while especially the nanofiber mats with lower areal weight—whether with additional mycelium or as pure membranes—tend to damages which clearly reduce the filtration efficiency. Although not in all the samples with unexpectedly low efficiency regarding the filtration of astra blue and chlorophyll, damages were visible, small undetected punctuations or imperfect alignment at the borders are a probable reason for such low filtration values.

For both smaller molecules, the nanofiber mats produced with the aforementioned electrospinning parameters are clearly not efficient as filters. This is similar to other reports of filtration of indigo carmine and other anionic dyes by PAN nanofiber mats in the literature [41,42]. The cationic dye safranin is usually tested according to adsorption instead of filtration and thus cannot be compared with the literature [43,44,45]. Astra blue and chlorophyll are normally not used as model molecules for filtration by nanofiber mats, so that our study serves as a base for future filtration experiments with these dyes.

The adsorption of the dye molecules on the filters varies not only with the dye, but also shows fluctuations which cannot be explained yet, but have to be tested further in future experiments.

## 4. Conclusions and Outlook

Needleless electrospinning was used to prepare nanofiber mats with areal weights of 0.9 g/m²–13.9 g/m². On the cut samples, mycelium of *P. ostreatus* and *P. ostreatus blue* was grown and partly thermally inactivated. Filtration tests in a pumped setup showed that filtration through these specimens was successful for the larger dye molecules under investigation, while the smaller ones were largely transported through the filters. Similar results were found in a diffusion cell setup. In both cases, the samples with very low filtration efficiency often showed obvious damages, underlining the importance of mechanically stabilizing nanofiber mats used for filtration purposes.

Further tests will include the investigation of the cycle stability for the thicker nanofiber mats, where tests will be performed without changing the filter in between to avoid apparent deviations in the filtration efficiency due to imperfect filter placement.

## Data Availability

All data obtained in this study are part of this paper.
